# Translating biosynthetic gene cluster architecture into biosensor design for microbial natural product discovery

**DOI:** 10.1042/EBC20250015

**Published:** 2026-08-19

**Authors:** Luisa M. Trejo-Alarcón, Víctor H. Tierrafría, Luis M. Salazar-García, Luz A. González-Salazar, Julio C. González-Aquino, Alfredo Martinez, Cuauhtémoc Licona-Cassani

**Affiliations:** 1 Tecnológico de Monterrey, Unidad de Biología Integrativa, The Institute for Obesity Research, Monterrey, Nuevo León, México; 2 Instituto de Biotecnología, Universidad Nacional Autónoma de México, Cuernavaca, Morelos 62210, México; 3 Tecnológico de Monterrey, Escuela de Ingeniería y Ciencias, Monterrey, Nuevo León, México

**Keywords:** antimicrobials, Biosensor, Biosynthetic Gene Cluster, drug discovery and design, high-throughput screening

## Abstract

Antimicrobial resistance (AMR) is a growing global health crisis, with mortality already in the millions and projections of up to 10 million deaths annually by 2050. Traditional discovery strategies, such as one strain many compounds-based screening of large strain collections, have yielded most of our current antibiotics but are slow, resource-intensive, and highly prone to rediscovery. In parallel, high-throughput sequencing has uncovered an enormous reservoir of biosynthetic gene clusters (BGCs), of which only a small fraction has been experimentally characterized, and many show extensive overlap in gene content. This combination of hidden diversity and functional redundancy demands new tools to prioritize, monitor, and rationally activate BGCs. In this review, we discuss how biosensors can be integrated with genome mining to accelerate antimicrobial discovery and BGC dereplication. We summarize the chemical scaffolds, biosynthetic logic and diagnostic enzymatic signatures of major antimicrobial classes, including β-lactams, tetracyclines, macrolides, aminoglycosides, glycopeptides, lincosamides, polyenes, and azoles, and highlighting representative biosensors that target each scaffold. The biosensors discussed use transcription factors, enzymes, aptamers, CRISPR systems and stress-response modules to generate specific and sensitive signals in complex matrices. We argue that translating BGC architecture into biosensor design creates a practical framework to rapidly discriminate from novel activities, but most importantly, to guide the exploration of chemical space around clinically important scaffolds in the era of escalating AMR.

## Introduction

Antimicrobial resistance (AMR) represents a global health crisis with serious social and economic consequences. Recent data show that ∼5 million people die each year from diseases associated with bacterial AMR and without effective measures, yearly AMR-related deaths could reach 10 million by 2050 [1]. Taken together, our need for development of new antibiotics is outpaced by rising AMR. In this context, the UN goal of reducing deaths associated with bacterial AMR by 10% by 2030 emphasizes both the scale of the challenge and the urgent need to accelerate the antimicrobial drug discovery pipeline [2].

Most of the antimicrobials currently available for human and animal use were discovered through bioactivity assays using large microbial collections [3]. The one strain many compounds (OSMAC) approach includes culturing thousands of strains under several culture conditions and using crude extracts and bioactivity assays to guide identification of compounds through successive purification rounds [4]. While time and resource-consuming, OSMAC remains one of the simplest and most effective strategies for drug discovery research.

The democratization of sequencing technologies has generated a volume of genomic data that far exceed our experimentation capabilities. A recent survey of more than 200,000 microbial genomes revealed that only approximately 3% of the biosynthetic diversity has been experimentally characterized [5]. In addition, databases are nourished by data generated under a strong experimental bias, as most efforts focus on characterizing a narrow subset of taxa [6]. Furthermore, large-scale biosynthetic gene clusters (BGCs) analysis reveals extensive overlap in biosynthetic gene content, implying a high risk of rediscovering the same compounds [7]. Systematic prioritization and the combination of throughput approaches are required for accelerating the success rate of discovery of novel compounds [8–12].

**Figure 1:**
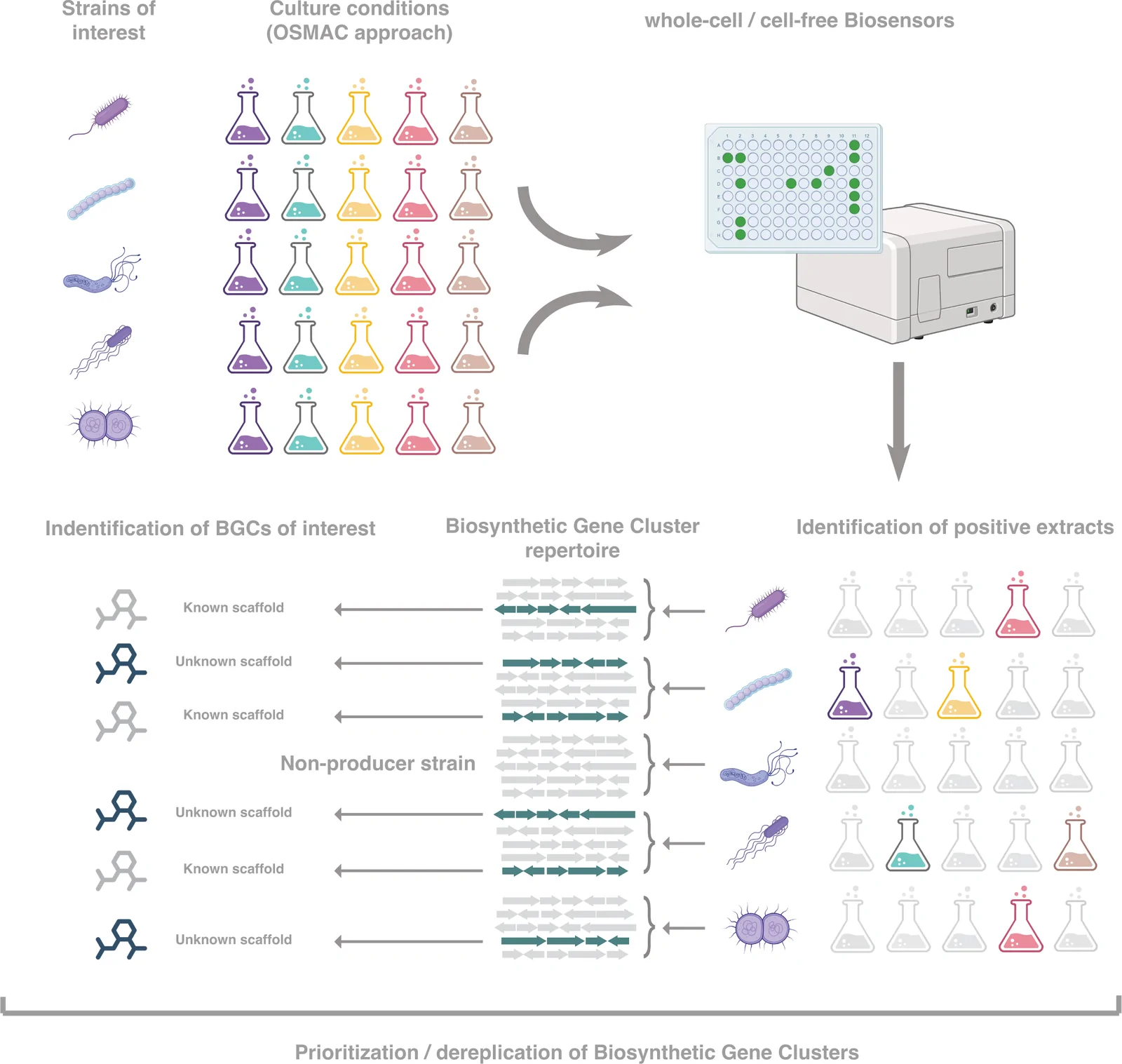
Proposal on how whole-cell or cell-free biosensors can be systematically integrated with genome mining to accelerate natural product discovery and dereplication

In this context, genome mining has emerged as a powerful *in silico* strategy to define and prioritize BGCs, changing the classical OSMAC approach towards rational identification of clusters with the highest biosynthetic relevance [13,14]. However, genome mining alone does not provide experimental signals. Biosensors become essential complementary tools as they allow for rapid detection, monitoring and optimization of BGCs expression [15]. In this review, we revise different biosensor assemblies implemented in both whole-cell and cell-free systems (CFS) and discuss how these tools can be integrated into the drug discovery pipeline to improve discovery and support BGC dereplication (Figure 1). Specifically, we outline and review the biosensors that target defined classes of BGCs, including β-lactams, tetracyclines, macrolides, aminoglycosides (AGs), glycopeptides, and lincosamides, while also considering their potential to uncover novel mechanisms of action and antimicrobial molecules.

## Biosensors: definition and composition

**Figure 2:**
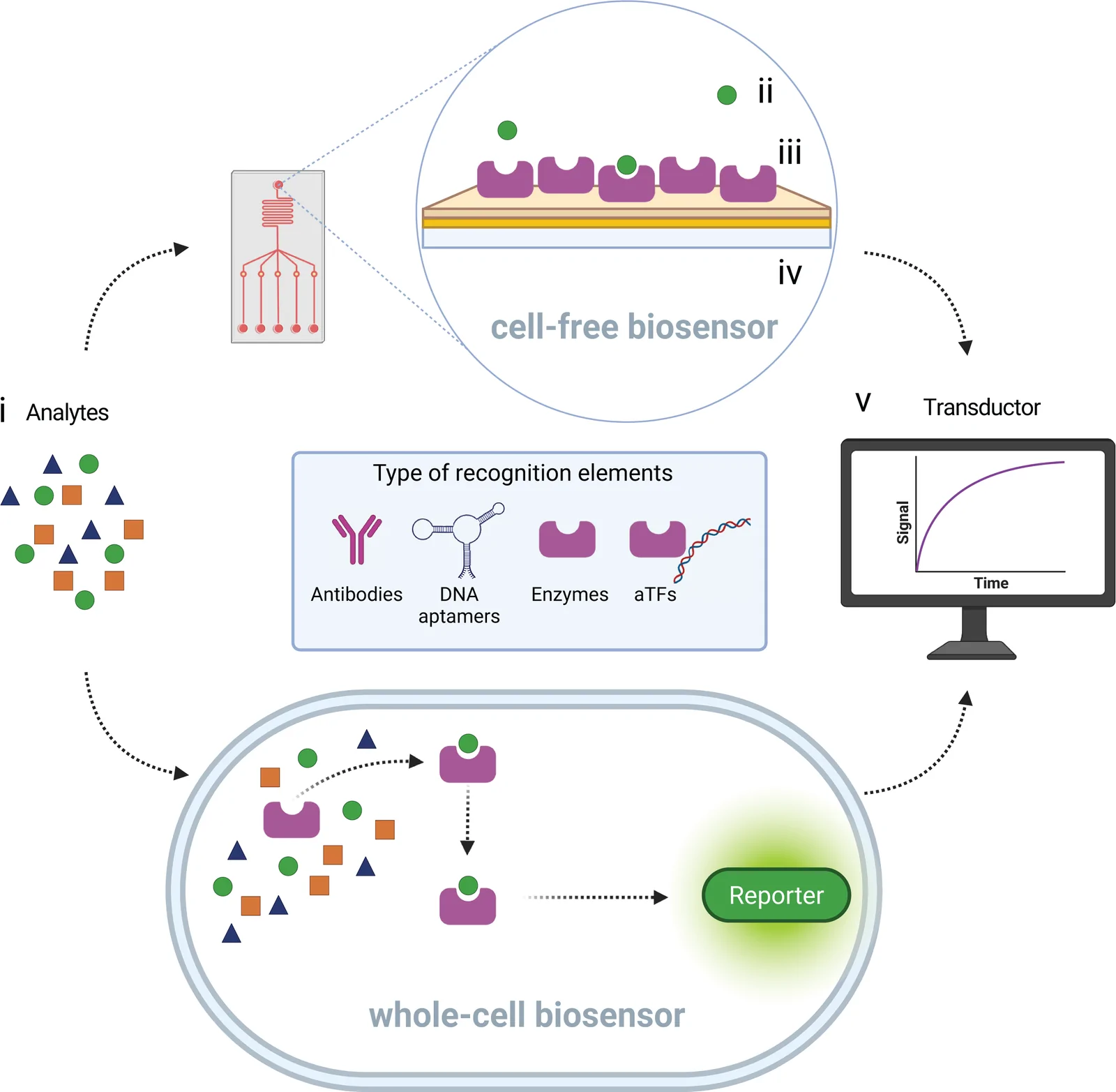
Key components of biosensors **Top panel:** Cell-free system (CFS). Simple representation of an electrochemical biosensor highlighting the recognition element (iii) and transducer material (iv) as fundamental parts. Although the sample (i), the target analyte (ii), and the output device (v) are not part of the biosensor, they are included for the sake of clarity on biosensor functioning. **Middle panel:** Additional recognition elements of biological origin that can be used in two designs are indicated in the long-rounded box. **Bottom panel:** Whole-cell system (WCS). The scheme denotes the functioning of an optical biosensor based on an aTF and a fluorescent reporter protein activating a positive feedback loop in response to the target analyte

There are two accepted definitions of biosensors. The first refers to self-contained devices or CFS that produce measurable signals in response to the presence of an analyte detected by a biological recognition element (Figure 2). A second definition, mostly used in synthetic biology, refers to whole-cell systems (WCS) that encode all the bioparts required to produce a measurable signal in response to a given analyte [16]. Whether CFS or WCS, biosensors comprise two core components: a recognition element that interacts with the analyte and a transducer that transforms that interaction into a physicochemical signal. In CFS, there is a third key component required to amplify the signal, reduce noise, and deliver the processed signal to an output device. This component is known as the signal processor (Figure 2) [16].

## Cell-free versus whole-cell derived biosensors: advantages and disadvantages

Cell-free and whole-cell biosensors have complementary advantages. CFSs are insensitive to analyte toxicity and free from intracellular regulation, which enables precise detection of highly toxic compounds such as heavy metals and antibiotics [17,18]. These features make CFS more suitable for discovery purposes, since high sensitivity and wide tolerance range are desirable features in a sensing device. Another advantage of CFSs is their portability as they do not need special maintenance or storage to keep optimal functionality. CFSs can be easily freeze-dried and fixed onto paper discs or other supports, stored without refrigeration and reactivated by simple rehydration [19]. In addition, CFSs are safe to work with parts from pathogenic microorganisms that makes them attractive for particular applications [20].

In contrast, WCSs make use of the full metabolic and regulatory machinery of a living cell. WCSs can integrate complex genetic circuits that can be configured for several purposes, including amplification of weak signals [21]. However, WCSs are prone to false positives due to cross-reactivity with endogenous metabolites and often operate within narrow pH and temperature ranges [22]. This apparent limitation can also be an advantage in applied settings, since WCSs are well suited for industrial processes in which screening is performed under physiological culture conditions. However, CFSs and WCSs can be used as complementary tools. While CFSs are practical for screening or detecting toxic compounds, WCSs provide robustness and versatility. In the context of antimicrobial discovery and BGC dereplication, a combination of both configurations could provide flexible and effective workflows for rapid initial screening and testing of highly toxic analytes for pathogenic targets.

## Biosensor composition: sensing elements, transducers, and chassis

Biosensors can be classified according to their components used for sensing and signal transduction. Based on the sensing element, biosensors can be whole-cell, biocomplementation and enzyme-based biosensors. Based on their transducer, biosensors can be classified as optical, electrochemical and mass biosensors [21,23]. Finally, the recognition elements include antibodies, proteins (enzymes or transcription factors TFs), and DNA aptamers. Transcription factors (TF)-based biosensors rely on the interaction with specific DNA fragments to complete the signal-response process.

## Biosensors for β-lactams derived biosynthetic gene clusters

The β-lactam ring is a chemical scaffold that is formed by a four-membered saturated cyclic amide ring [24,25]. For the specific case of β-lactam antibiotics, bioactivity is derived from inhibiting the activity of enzymes responsible for cross-linking peptidoglycan strands during the process of cell wall synthesis. Biosynthesis of these molecules is encoded by BGCs that are grouped into two pathways. In the classical pathway, the condensation of the tripeptide L-α-aminoadipic-L-cysteine-L-valine is catalyzed by ACVS (*pcbAB*) and its subsequent cyclization by isopenicillin N synthase (*IPNS/pcbC*). The alternative pathway is specific for carbapenem and clavam-derived molecules in which β-lactam synthetase (β-LS) generates the β-lactam ring [26,27]. All β-lactam-derived BGCs include tailoring enzymes and regulatory elements that give rise to an extensive diversity of chemistry [28,29]. In this context, genome mining of β-lactam BGCs provides a framework for rationally determining where to search for new compounds and how to detect their production. Complementarily, biosensors can be used as rapid screening tools to optimize production and explore analogs generated through combinatorial biosynthesis.

A variety of enzyme-based and whole-cell biosensors have been developed for β-lactam detection and bioprospection (Table 1) [30–34]. A cell-free biosensor based on a class A β-lactamase with reduced hydrolytic activity binds fluorescein and detects the presence of penicillins and cephalosporins β-lactam-derived molecules at sub-micromolar concentrations [32]. Another biosensor reported in *Bacillus subtilis* uses heterologous expression of the *Staphylococcus aureus* BlaR1/BlaI system. Upon the presence of a β-lactam derived analyte, the product of *blaZ* gene is secreted to inactivate the β-lactam antibiotic. Replacement of *blaZ* by a lux operon *lux*ABDCDE yielded a luminescent whole-cell biosensor that responded to ten β-lactams from four structural classes with very high sensitivity and selectivity [31]. In *S. aureus*, a *murZ*^p^-*lacZ* fusion system was developed for the discovery of antibacterial inhibitors targeting cell-wall biosynthesis. The biosensor was used to screen >9000 natural product extracts, and identified one potential analyte that was not detected by other methods [30]. Complementary designs in *Streptomyces* include a whole-cell biosensor that uses VanS/R two-component system promoters fused to *luxCDABE* to identify 17 candidates of cell-envelope-targeting antibiotics [33].

**Table 1:** Description of whole-cell and cell-free biosensors included in the present review. Biosensors were classified according to different groups including biosensor configuration, host platform, type of analyte, recognition element/entity, signal transdcutor and signal type.

Biosensor type	Host platform	Natural product target	Analytes	RE type	RE entity	Signal transductor	Signal type	Limit of detection	Refs.
		β-lactams	Penicillins and cephalosporines	Enzyme		Optical	Fluorescence	0.05 μM	[32]
		β-lactams	Carbenicillin, cephalosporin C and cefotaxime			Optical	Luminescence	ng/l range	[31]
Whole-cell	*Staphylococcus aureus*	β-lactams	Cell-wall biosynthesis inhibitors					NA	[30]
Whole-cell	*Streptomyces*	β-lactams	Vancomycin, bacitracin, flavomycin, and polymyxin E			Optical	Luminescence	NA	[33]
Whole-cell		β-lactams	Polymyxin B	TF		Optical	Fluorescence	0.125 and 12 μg/ml	Yin et al., 2022 [34]
Whole-cell	*Shewanella oneidensis*	β-lactams	Penicillin G and carbenicillin	TF		Optical	Luminescence	10 μg/ml	Yin et al., 2022 [35]
Whole-cell	*Shewanella oneidensis*	β-lactams	Penicillin G and carbenicillin	TF		Optical	Fluorescence	10 μg/ml	Yin et al., 2022 [35]
WCB	*Escherichia coli K-12*	Tetracyclines	Tetracycline, oxytetracycline, chlortetracycline, methacycline, doxycycline, demeclocycline, and minocycline	TF	TetR	Optical	Luminescence	pM range	[38]
WCB	*Escherichia coli*	Tetracyclines	Tetracycline	aTF	TetR	Optical	Fluorescence	0.1 ng/ml	[43]
WCB	*Escherichia coli DH5a*	Tetracyclines	Tetracycliene, oxytetracycline, chlortetracycline, deoxytetracycline, minocycline, and methacycline	TF	TetR	Optical	Fluorescence (mCherry)	5.32–7.85 μg/kg	[41]
WCB	*Escherichia coli DH5a*	Tetracyclines	Tetracycliene, oxytetracycline, chlortetracycline, deoxytetracycline, minocycline, and methacycline	TF	TetR	Optical	Fluorescence (GFP)	7.58–10.2 μg/kg	[41]
CFS	NA	Tetracyclines	Tetracycline	Enzyme	TetX2	Optical	Colorimetric	60 nM	[48]
CFS	NA	Tetracyclines	Tetracycline	Enzyme	TetX2	Electrochemical		40 nM	[48]
		Tetracyclines	Tetracycline, oxytetracycline, and doxycycline	DNA aptamer		Optical	Fluorescence	0.1–0.7 nM	[44]
		Tetracyclines		DNA aptamer		Electrochemical		0.20 nM in milk	[45]
		Tetracyclines	Tetracycline, doxytetracycline, oxytetracycline	TF		Optical	Fluorescence	NA	[18]
CFS		Tetracyclines	Anhydrotetracycline, oxytetracycline, doxycycline, and chlortetracycline	aTF	TetR	Optical	Fluorescence	0.5 uM	[47]
CFS		Tetracyclines	Anhydrotetracycline, oxytetracycline, doxycycline, and chlortetracycline	aTF	TetR	Optical	Fluorescence	0.01 uM	[47]
		Macrolides	Erythromycin, azithromycin, clarithromycin	TF		Optical	Fluorescence	NA	[18]
		Macrolides	Erythromycin, clarithromycin and roxithromycin	TF				NA	[92]
WCB		Macrolides	Erythromycin, oleandomycin, narbomycin, picromycin, clarithromycin, and azithromycin	aTF	MphR	Optical	Luciferase	NA	[93]
WCB		Macrolides	Erythromycin, clarithromycin, roxithromycin, and azithromycin	aTF	MphR	Optical	Fluorescence	13 nM for erythromycin	[95]
CFS		Macrolides	Erythromycin, roxithromycin, azithromycin, and clarithromycin	aTF	MphR	Optical	Fluorescence	0.1–0.5 uM	[47]
CFS		Macrolides	Erythromycin, roxithromycin, azithromycin, and clarithromycin	aTF	MphR	Optical	Fluorescence	0.01 uM	[47]
WCB	HeLa and HEK293T cells	Macrolides	Rapamycin	Heterodimeric binding protein	FKBP12-FRB	Optical	Fluorescence	0.1 nM	[96]
CFS		Macrolides	FK506 (tacrolimus)	Tacrolimus binding protein	FKBP1A	Optical	Fluorescence	3 ng/ml	[97]
CFS		Anthracyclines	Daunorubicin	DNA aptamer		Electrochemical		0.3 nM	[98]
CFS		Peptides	Actinomycin D	DNA		Optical		33.3 nM	[99]
CFS		Anthracyclines	Nogalamycin	DNA		Optical		0.143 uM	[99]
CFS		Anthracyclines	Doxorubicin	DNA		Optical		1.11 uM	[99]
CFS		Anthracyclines	Daunomycin	DNA		Optical	Monochromatic polarized light	0.5 uM	[99]
		AGs				Optical	Luminescence	NA	
		AGs	Gentamicin, geneticin			Optical	Fluorescence	NA	[61]
		AGs	Kan, Neo, Tob, DH-Str, Gen, Spe, Str, Ami, Apr, Par	DNA aptamer	ordered mesoporous carbon (OMC)	Electrochemical		2.47 nM	[63]
		AGs	Kan, Neo, Tob, DH-Str, Gen, Spe, Str, Ami, Apr, Par	DNA aptamer	OMC and Ti3C2 MXene	Electrochemical	Differential pulse voltammetry	3.51 nM	[23]
Whole-cell	*Shewanella oneidensis*	Glycopeptides	Vancomycin	TF		Optical	Luminescence	10 μg/ml	Yin et al., 2022 [35]
Whole-cell	*Shewanella oneidensis*	Glycopeptides	Vancomycin	TF		Optical	Fluorescence	10 μg/ml	Yin et al., 2022 [35]
		Glycopeptides	Vancomycin			Optical	Fluorescence	2.79 ng/ml	[68]
		Lincosamides	Lincomycin	Aptamer		Electrochemical/ optical	Luminescence	1.6 × 10^-13^ mol/l	[74]
		Polyenes	Amphotericin B	TF				NA	[79]
		Azoles		DNA aptamer				NA	[77]
		Azoles	Tebuconazole	Luciferase		Optical		NA	
		Anticancer		Caspase sensor protein		Optical	Fluorescence	NA	[100]
		Anticancer	Bleomycin	Enzyme		Photoelectrochemical		0.5–500 nM	[69]
			Type I interferon inhibitors	Enzyme		Optical		[NA]	Kinder et al., 2020 [105]
			Rapamycin			Optical		0.1 nM	Lee et al. [96]

Stress-response mechanisms further complemented the repertoire of biosensors for β-lactam-derived molecules. A σ^M^-EGFP reporter in *B. subtilis* shows dose-dependent fluorescence in response to polymyxin B and related β-lactam analytes with a detection range between 0.125 and 12 μg/ml [34]. In *Shewanella oneidensis*, a biosensor was developed based on the PghKR two-component system (which senses peptidoglycan damage). A *blaA*^p^ fusion with either *luxCDABE* (luminescence) or *sfgfp* (fluorescence), showed a dose-response signal to peptidoglycan-targeting analytes (β-lactams, vancomycin, D-cycloserine) with high sensitivity (concentrations of 10 μg/mL) [35].

## Biosensors for the detection of tetracyclines

Tetracyclines are a subtype of polyketides with a linear, fused tetracyclic nucleus that can generate a wide range of chemical structures [36,37]. Tetracyclines are mainly produced by *Streptomyces* species and function by inhibiting protein synthesis upon blocking the attachment of the aminoacyl-tRNA to the ribosomal receptor in the bacterial 30S ribosomal subunit [38].

Tetracycline-BGCs are formed mainly by a type II polyketide synthase (PKS) that generates a linear poly-β-keto backbone followed by a characteristic OxyK/OxyN/OxyH/OxyI cyclization ‘tetracycline signature’ [39]. A wide range of FAD-dependent oxidoreductases, cytochrome P450, glycosyltransferases, methyltransferases, and KAS III synthases diversifies the structural core [40]. From a biosensor perspective, this shared cyclization signature provides a practical DNA/protein marker for targeting tetracycline-like pathways during genome mining and supporting dereplication of redundant BGCs in discovery workflows [40].

A broad set of TetR-based whole-cell biosensors has been developed by fusing TetR-tetA^p^ regulatory modules to different reporters (Table 1). In *E. coli* K-12 and DH5α, TetR-tetA^p^ fusions controlling the *lux* operon,GFP or mCherry have been used to detect panels of tetracyclines in soils and other samples with high sensitivity [38,41]. TetR has also been integrated into an epigenetic memory circuit based on DNA methylation by replacing the AraC-arabinose trigger with a TetR-tetracycline module [42]. In this design, a two-plasmid whole-cell biosensor can ‘record’ transient exposure to tetracycline [42,43].

Beyond transcriptional whole-cell biosensors, several cell-free platforms extend tetracycline detection in food and water matrices (Table 1). DNA aptamers such as OTC5, either in fluorescence mode or as ferrocene-labeled aptasensors on electrochemical electrodes, achieve sub-nanomolar detection of tetracycline, oxytetracycline, and doxycycline in complex samples [44,45]. TetR-controlled CFS regulating Cas12a arrays or Broccoli RNA (3WJdB) convert tetracycline binding into CRISPR collateral cleavage or RNA-dye fluorescence [46,47]. Finally, enzyme-based biosensors built around TetX2 monooxygenase (conversion of tetracycline to 11-a-hydroxytetracycline) and the redox dye thionine support both electrochemical and colorimetric signals, detecting tetracycline at 40–60 nM [48].

Together, these efforts show how regulatory proteins, aptamers, CRISPR components, and tailoring enzymes can be repurposed as targets for the design of biosensors. This is particularly relevant given that third-generation tetracycline development is focused on a combination of semisynthesis and total synthesis [49], making it feasible to redirect the detection strategies described here from monitoring of contaminants towards systematic drug discovery and guided expansion of the tetracycline scaffold.

## Biosensors for aminoglycosides

AGs are cationic oligosaccharides with an aminocyclitol core substituted with multiple amino and hydroxyl groups. AGs bind to the 16S rRNA in the 30S ribosomal subunit, affecting the decoding site and promoting mistranslation, leading to inhibition of protein synthesis [50]. Interestingly, the same ribosomal interaction can also induce codon readthrough (CR) at premature termination codons, a phenomenon that has been exploited to explore AGs as candidates for treating genetic diseases such as cystic fibrosis and Duchenne muscular dystrophy [51–53].

AGs BGCs fall into three major types according to the central aminocyclitol: 2-deoxystreptamine (2DOS), myo-inositol-1-phosphate (MIP), and fortamine/2-deoxifortamine [54,55]. 2DOS-derived AGs (neomycin, kanamycin, gentamicin, butirosin, tobramycin) share a conserved seven-enzyme core (NeoC, NeoS, NeoE, NeoM, NeoD, NeoQ, NeoB) [55]. Structural diversification is achieved by cluster-specific tailoring enzymes, such as AHBA-pathway genes in butirosin [56], SAM-dependent methyltransferases in gentamicin [57], or deoxygenases AprD4/AprD3 in tobramycin/apramycin [53]. MIP-derived clusters such as streptomycin encode characteristic enzymes (StsC, StrB1, StrD/StrE/StrM) [58], while fortimicin/istamycin clusters derive from fortamine/2-deoxifortamine. Many AG BGCs also include conserved G/H/I regulatory triplets proposed as sensor-response modules, which are attractive targets for biosensor construction and genome mining.

A dual-luciferase CR reporter was designed by expressing Renilla and Firefly luciferases in the same reading frame separated by a premature stop codon. AG-induced readthrough restores Firefly signal, providing a quantitative measure of CR (Table 1) [59,60]. Using a similar design, a dual-fluorescent system in *Saccharomyces cerevisiae* with EmRFP and yEGFP separated by stop codons, was used to quantify CR in response to gentamicin and geneticin, offering a yeast platform for screening new analogs [61,62]. An aptamer-based electrochemical biosensor using DNA aptamers immobilized on ordered mesoporous carbon-modified screen-printed electrodes was used to detect a broad panel of AGs in milk [63]. Finally, a paper-based device incorporating a T3 RNAP-dependent *in vitro* transcription/translation system measures inhibition of NanoLuc synthesis by AGs in serum or whole blood samples (Table 1) [64].

Taken together, the clear enzymatic signatures of AG BGCs and the diverse AG biosensors—CR reporters, aptamer electrodes, and paper devices, offer complementary tools to (i) detect AG activity, (ii) link that activity back to specific biosynthetic pathways, and (iii) support dereplication and discovery of novel AG molecules.

## Biosensors for glycopeptides and lipoglycopeptides

Glycopeptides are glycosylated cyclic or polycyclic non-ribosomal peptides with defined sugar moieties. Glycopeptides inhibit bacterial cell-wall synthesis by binding the D-Ala-D-Ala end of peptidoglycan precursors, blocking cross-linking and weakening the wall, leading to cell lysis. Clinically, glycopeptides and lipoglycopeptides are used mainly against Gram-positive pathogens, including problematic *Staphylococcus* and *Streptococcus* strains.

Glycopeptide-derived BGCs can be grouped into five main types (I–V) according to their chemical structure and mechanism of action. Vancomycin-type and teicoplanin-type glycopeptides differ in their heptapeptide sequence and aromatic building blocks, whereas type V glycopeptide-related peptides show stronger structural divergence. Glycopeptide BGCs typically contain three major gene sets, including a seven-module NRPS core, biosynthetic genes for non-proteinogenic amino acids (such as Dpg, Hpg and Bht), and a suite of tailoring, resistance, and regulatory genes, including P450 monooxygenases, glycosyltransferases, sulfotransferases, and regulatory genes [65]. Genome mining therefore centers on detecting the characteristic seven-module NRPS, the non-proteinogenic-precursor pathways, and the pattern of tailoring enzymes to predict cross-linking, glycosylation, and sulfation and to flag clusters with high potential for structural novelty [66,67].

Mu and collaborators developed a dual-emission fluorescence biosensor that combines a peptide conjugated to blue-emitting aggregation-induced emission luminogens (AIEgens) with a red-emitting vancomycin aptamer-gold nanocluster construct (aptamer-AuNCs) (Table 1). The sensor discriminates vancomycin from other glycopeptides in complex matrices [68]. Additionally, Kong et al. created a photoelectrochemical biosensor for the detection of the anticancer glycopeptide bleomycin. The sensor uses a tungsten sulfide nanorod array (WS_2_NA) electrode modified with gold nanoparticles and a silver nanoparticle/flake-like zinc metal-organic structure nanozyme with peroxidase-mimetic activity as the sensing matrix, and it operates over a dynamic range of 0.5–500 nM [69].

## Biosensors for lincosamides

Lincosamides are antibiotics with an amino-octose (eight-carbon) sugar linked via an amide bond to an L-proline moiety, which serve as the core chemical scaffold. Lincosamides bind to the 50S ribosomal subunit and block the transpeptidation step of protein synthesis in Gram-positive pathogens. The BGCs of lincosamides display a hybrid, thiol-dependent architecture [70].

Typical lincosamide BGCs, such as lincomycin and celesticetin, encode an eight-carbon sugar bearing a mercapto group at C1, linked to L-proline through an amide bond [71]. Lincosamide BGCs contain distinctive enzymatic activities, including Ntn-hydrolases such as LmbA, which mediates C-C cleavage to generate the 4-alkyl-L-proline unit, and thiol-dependent glycosyltransferases LmbT and LmbV, which couple glycosylation to low-molecular-weight thiols such as ergothioneine and mycothiol [72]. Together with PLP-dependent enzymes that process cysteinyl intermediates, homologs of LmbA, LmbT, and LmbV provide a practical marker set for tracking new lincosamide BGCs and anticipating variation in S-functionalization and in the 4-alkyl-L-proline fragment [73].

Biosensors for lincosamides include an electrochemiluminescence biosensor that relies on the interaction between an aptamer and a molecularly imprinted polymer for lincomycin (Table 1) [74]. When lincomycin is present in the sample, it binds to the aptamer. This aptamer-lincomycin complex prevents the polymer from binding to lincomycin. When a voltage is applied to the biosensor, electrochemical luminescence is generated. The amount of luminescence generated depends on the quantity of lincomycin present in the sample. This biosensor has the potential to be used to develop new antibiotics and to detect antibiotic resistance.

## Biosensors for polyenes and azoles

Polyenes class of antifungals bind sterols and form pores [75], whereas azoles inhibit ergosterol synthesis [76]. These mechanisms of action have been used in a *B. subtilis* whole-cell biosensor using the LnrJK two-component system and the PlnrL231 promoter to detect amphotericin B (Table 1). For azoles, SELEX-derived aptamers and a yeast nanoLuc reporter strain responsive to tebuconazole enable specific detection in spiked samples at low μg/l levels [77,78]. Together, these designs illustrate how antifungal-specific targets can be converted into practical biosensor signals [79].

## Biosensors for macrolides

Macrolides are macrocyclic lactones (cyclic carboxylic esters) with a 12–16-member ring size that typically display antibiotic activity by inhibiting protein synthesis. Macrolactones of 17–18 members are also considered macrolides and generally affect the function of RNA polymerase rather than the ribosome [80]. Macrolides, which inhibit protein synthesis bind to the nascent peptide exit tunnel of the 50S ribosomal subunit providing broad-spectrum antimicrobial activity [81,82].

At the BGC level, macrolides can be grouped into (i) ‘classical macrolides’ and polyenes (e.g. nystatin, amphotericin B, pimaricin, candicidin), produced by modular Type I PKS, sugar-decorating enzymes (glycosyl transferases), P450 oxidases and ABC transporters [83,84]; (ii) β-amino acid macrolactams generated by hybrid PKS-NRPS (Non-Ribosomal Peptide Synthase) pathways, therefore encoding β-amino acid biosynthesis (e.g. glutamate mutases, decarboxylases), adenylases, acyltransferases, and acyl-carrier proteins [85,86]; and (iii) polycyclic tetramate macrolactams (PTMs) that rely on compact-iterative PKS–NRPS systems [87], together with dehydrogenases and P450 enzymes for cyclization of the rings [88–90]. Based on the BGC structure, certain enzymes such as VinN/VinM/VinK serve as markers for β-amino acid macrolactams [91], while genes like *ftdB* identify PTMs; in addition, multi-layered regulators and two-component systems for these clusters (e.g.* garR1–garR7*) provide a logic starting points for biosensor design [91].

Several macrolide biosensors built based on the macrolide-responsive transcription factor macrolide phosphotransferase regulator (MphR) have been developed and can be directly linked to BGC dereplication and targeted genome mining discovery (Table 1). In mammalian cells, one of the first biosensors combines the *E. coli* macrolide repressor (E) and the operator (ETR) derived from the erythromycin-resistance regulon. In the system called E.REX, the E_OFF_ regulation is a fusion protein of the erythromycin resistance gene repressor E (MphR(A)) and the herpes simplex virus transactivation domain (ET1) and an ET-dependent macrolide-responsive promoter (PETR), assembled from the *E. coli* MphR(A)-specific operator (ETR) and a minimal promoter derived from the human cytomegalovirus promoter [92]. The same sensing logic was used in a versatile *in vitro* assay in which antibiotic sensor proteins (including MphR) bind cognate DNA attached to a solid surface; the changes in binding are converted into an immuno-based colorimetric signal that detects macrolides and tetracyclines at ng/ml levels [92].

Möhrle et al. constructed a robust *E. coli* whole-cell biosensor based on the *mph(A)* operon (*mph*, *mrx*, and *mphR*). In this design, the native mph promoter, normally repressed by MphR(A) in the absence of macrolides, was fused to the *luxCDABE* operon from *V. fischeria*. To improve the background signal, the strain Tf481A was engineered with an outer-membrane permeability defect to hydrophobic antibiotics and overexpression of MphR(A). The biosensor generated strong luminescence in response to erythromycin and second-generation derivatives (clarithromycin and azithromycin), oleandomycin, narbomycin, picromycin, but not to unrelated antibiotics, and the signal was robust across agar and microplate formats, making it well suited for rapid dereplication and identification of macrolides in crude extracts [93]. Using a similar design, Miller et al. redesigned an MphR-based *E. coli* sensor by fusing the mph promoter and *mphR* to superfolder green fluorescent protein (sfGFP) and placing the macrolide phosphotransferase gene *mphA* under a constitutive promoter to trap phosphorylated macrolides inside the cell [94,95]. Collectively, these macrolide biosensors illustrate how MphR(A)-centered designs can serve as sensitive, macrolide-focused tools for dereplication, bioprospecting, and guided exploration of macrolide BGC diversity.

## Biosensors for other non-antimicrobial macrolides

Non-antimicrobial macrolides such as rapamycin and FK506 have also inspired highly specific biosensor designs (Table 1). For rapamycin, an optical whole-cell biosensor in eukaryotic cells uses rapamycin-binding domains, an intein and a fluorescent nuclear localization tag that reassemble only in the presence of the drug, giving sensitive nuclear fluorescence signals [96]. For FK506, a cell-free optical biosensor employs the human receptor FKBP1A fused to GFP, in which binding of FK506 is transduced into a fluorescent signal in complex samples [97]. Both systems were built for therapeutic drug monitoring but can be adapted to screening crude extracts and linking signals to macrolide BGCs.

## Biosensors for the detection of anthracyclines with anticancer bioactivity

Anthracyclines and other anticancer agents bind DNA by intercalating their core structure (anthraquinone ring) between base pairs. For anthracyclines such as daunorubicin, doxorubicin, and nogalamycin, DNA-intercalation has been exploited in electrochemical biosensors using double-stranded-DNA-coated, nanotube-modified electrodes (Table 1). In these biosensors, changes in electrochemical behavior generate signals upon analyte binding in serum or urine, and in surface plasmon resonance sensors with immobilized oligonucleotides that yield reliable affinity constants [98,99]. In addition to anthracyclines, high-throughput FRET-based assays in HeLa cells expressing caspase sensor proteins detect apoptosis induction by natural-product libraries, enabling rapid identification of non-anthracycline anticancer leads [100]. Together, these DNA- and cell-based systems illustrate how conserved pharmacological mechanisms can be repurposed as generic recognition principles for the discovery and dereplication of anticancer compounds.

## Biosensors beyond BGC dereplication: applications for the discovery of natural products

Biosensors can support the discovery of novel chemical classes or reveal new mechanisms of action in previously known compounds. For example, a biosensor based on M. tuberculosis cytochrome P450 (CYP) was used to screen a library of 140 known compounds known for their ability to inhibit CYP activity in humans. This target-based biosensor enabled the discovery of two CYP121 inhibitors with strong antimycobacterial activity and lower toxicity than the reference compound econazole [101]. A complementary approach was used to screen a library of 14,000 compounds for the discovery of novel antimicrobial agents [102]. Authors designed a WCB in *B. subtilis* of the firefly luciferase gene fused to promoters of genes from DNA, RNA, protein, cell wall and fatty acid biosynthetic pathways [102]. found at least seven hits with unknown mechanisms of action, especially with *yheI* and *yorB* biosensors. These examples show that high-throughput screening using biosensors are not limited to detecting known scaffold classes but can also expose hidden bioactivities in existing chemical space.

If the goal is to discover entirely new chemical classes, biosensors can be integrated in bioprospecting workflows [11]. These workflows start with the isolation and genomic characterization of microbes from underexplored environments to increase the odds of discovering chemical structures never seen before [12,103]. Genomic metrics such BiNI [10] can then be used to prioritize strains with high novelty and biosynthetic potential, which are subsequently cultured under multiple conditions to maximize expression of cryptic BGCs. In those cases, the use of broad-spectrum biosensors would rapidly narrow the number of extracts with the relevant bioactivity. Further metabolo-genomics analysis would help to determine the structures of active compounds. Because the producer strains and their BGCs are tracked throughout the process, these bioprospecting workflows facilitate both dereplication and the discovery of genuinely novel natural products.

Another example of discovery of novel compounds is shown by Xu et al., where a fluorescence-based whole-cell biosensor in *Streptomyces albus* J1074 was used to rapidly report the expression of cryptic BGCs [104]. Rather than testing numerous culture conditions, the authors used chemical elicitors added to a basal medium and placed promoters from transcription factors associated with cryptic BGCs upstream of a reporter gene, so that only cells in which elicitors activated silent clusters became fluorescent. This strategy provided a practical high-throughput route to identify cluster-activating conditions and prioritize strains for natural product discovery.

In future applications, biosensors may contribute not only to discovery and dereplication, but also to the standardization and automation of screening workflows. Bioinformatic pipelines can be designed to continuously calculate genomic novelty and biosynthetic potential metrics, as new genomes are rapidly incorporated into global databases, supporting strain prioritization in real time. In parallel, selected strains can be screened in high-throughput formats under arrays of culture conditions or chemical elicitors using liquid-handling platforms, while customized scripts process the signals to identify positive hits for downstream metabolomic and genomic characterization. Although such automation requires initial implementation, it offers clear advantages in traceability, robustness and scalability, and can strengthen the systematic search for new antimicrobial compounds together with their producer strains and BGCs.

## Summary

- AMR, and in general the drug discovery pipeline, demands smarter approaches, not just more discovery.
- For several reasons, genome mining reveals rich but redundant biosynthetic space among the natural product microbial producers.
- Biosensors provide pathway-specific, scalable readouts for laboratory experimentation.
- BGC architecture can be translated into biosensor design.
- Biosensors can be used for prioritization and dereplication of specific classes of BGCs and this can be integrated into workflows for guided drug discovery.

## Open Access

Open access for this article was enabled by the participation of Universidad Nacional Autónoma de México in an all-inclusive Read & Publish agreement with Portland Press and the Biochemical Society.

## Abbreviations

AG: Aminoglycoside
AMR: Antimicrobial resistance
BGC: Biosynthetic gene cluster
CFS: Cell-free system
CR: Codon readthrough
MIP: Myo-inositol-1-phosphate
OSMAC: One Strain Many Compounds
PKS: Polyketide synthase
sfGFP: Superfolder green fluorescent protein
TF: Transcription factor
WCS: Whole-cell system
2DOS: 2-Deoxystreptamine
PTM: Polycyclic tetramate macrolactam
